# Phylogenetic analysis of rabbit haemorrhagic disease virus (RHDV) strains isolated in Poland

**DOI:** 10.1007/s00705-017-3476-0

**Published:** 2017-07-12

**Authors:** Andrzej Fitzner, Wieslaw Niedbalski

**Affiliations:** Department of Foot and Mouth Disease, National Veterinary Research Institute, Wodna 7, 98-220 Zduńska Wola, Poland

**Keywords:** Rabbit haemorrhagic disease virus, Complete nucleotide sequence, Phylogenetic analysis

## Abstract

The aim of this study was to characterise the nucleotide and amino acid sequence of complete genomes (7.5 kb) from RHDV strains isolated in Poland and estimate the genetic variability in different elements of the viral RNA. In addition, the sequence of Polish RHDV isolates isolated from 1988-2015 was compared with the sequences of other European RHDV, including the RHDVa and RHDV2/RHDVb subtypes. The complete sequence was developed by the compilation of partial nucleotide sequences. This sequence consisted of approximately 7428 nucleotides. For comparison of nucleotide sequences and the development of phylogenetic trees of Polish RHDV isolates and reference RHDV strains representing the main phylogenetic groups of classical RHDV, RHDVa and RHDV2 as well as the non-pathogenic rabbit lagovirus RCV, the BLAST software with blastn and MEGA6 with neighbour-joining method was applied. The complete nucleotide sequence of Polish isolates of RHDV has also been entered into GenBank. For comparative analysis, nineteen complete sequences representing the main RHDV genetic types available in GenBank were used. The results of phylogenetic analysis of Polish RHDV strains reveals the presence of three classical RHDV genogroups (G2, G4 and G5) and an RHDVa variant (G6). The oldest RHDV isolates (KGM 1988, PD 1989 and MAL 1994) belong to genogroup G2. It can be assumed that the elimination of these strains from the environment probably occurred at the turn of 1994 and 1995. Genogroup G2 was replaced by the phylogenetically younger BLA 1994 and OPO 2004 strains from genogroup G4, which probably originated from the G3 lineage, represented by the Italian strains BS89. The last representatives of classical RHDV in Poland are isolates GSK 1988 and ZD0 2000 from genogroup G5. A single clade contains the Polish RHDV strains from 2004-2015 (GRZ 2004, KRY 2004, L145 2004, W147 2005, SKO 2013, GLE 2013, RED1 2013, STR 2012, STR2 2013, STR 2014, BIE 2015) identified as RHDVa, which clustered into genogroup G6, as represented by the RHDV strain Triptis 1996. All recent isolated RHDV isolates belong exclusively to RHDVa and no RHDV2 was diagnosed in Poland.

Rabbit haemorrhagic disease (RHD) is a highly contagious and often lethal viral infection of wild and domestic rabbits (*Oryctolagus cuniculus*), which first emerged in China in 1984 [19]. Over the next years RHD spread throughout the world affecting many countries, mainly in Asia and Europe but also in Africa, Central and North America, Australia and New Zealand [1]. The etiological agent, rabbit haemorrhagic disease virus (RHDV) is an icosahedral, non-enveloped, single-stranded positive-sense RNA virus of approximately 7437 nucleotides with two open reading frames (ORFs). The longer ORF1 (7034 nucleotides) encodes seven non-structural viral proteins p16, p23, p37 (helicase), p29, virus protein-genome linked (VPg), p15 (3C-like proteinase), p58 (RdRp) and a structural capsid protein-viral protein (VP60). The shorter ORF2 (353 nucleotides) encodes viral protein VP10 which, so far, has an unknown function [25]. It is well known that RNA viruses are characterized by their great potential for variability; however, RHDV strains isolated at the beginning of epizootic were conservative in terms of antigenic and genetic features regardless of the time and place of isolation. The first significant changes in the properties of the virus related to the lack of haemagglutination capacity in some RHDV strains [14]. In 1996, the occurrence of a new RHDV virus - the RHDVa variant - was reported in Italy and Germany [5, 27] and a non-pathogenic calicivirus (RCV) related to was also diagnosed in rabbits [4]. In 2010, a new RHDV subtype, designated as RHDV2, genetically and antigenically different from the classical RHDV and RHDVa variant, spread in Europe [7, 17, 18]. To explain the origin and the emergence of the new RHDV variant two hypotheses were proposed: 1) evolution from pre-existing non-pathogenic viruses circulating in Europe, and 2) a species jump [1]. On the basis of phylogenetic relationships, the pathogenic RHDV strains have been divided into three distinct groups: classical RHDV with the genogroups G1-G5 [26], the antigenic variant RHDVa (genogroup G6) [14], and the new subtype RHDV2 [16]. Distribution of RHDV into several genetic groups was developed on the basis of a similar time of virus isolation, regardless of the geographical distribution of disease [16]. The oldest RHDV isolates belong to the groups G1-G3. The representative of G1 group is RHDV strain AST89 isolated in Spain in 1989. The German strain FRG89 - the first sequenced RHDV and the Czech strain V351, introduced in 1995 in Australia and New Zealand to control the populations of wild rabbits, are representatives of the genetic group G2. The Italian RHDV strain BS89 isolated in 1989 and the German vaccine strain Meiningen are clustered into group G3. Genogroups G4 and G5 comprise many European RHDV from the 90s onwards, as well as the beginning of 21st century. All known strains of RHDVa variant are clustered into genogroup G6 [18].

In Poland, the first cases of RHDV were confirmed in 1988 [13]. In the following years, the disease spread rapidly and caused significant losses in rabbitries, regardless of race and type of breeding rabbits. In 1994, a non-haemagglutinating RHDV strain, defined as BLA, after the place of isolation (Blaszki village) was recognized, and then in middle of the first decade of the 21st century, RHDV strains exhibiting features similar to the RHDVa variant were isolated in Poland [6, 9]. To date, no RHD outbreaks caused by the RHDV2 subtype were confirmed in Poland. The main reason for this could be a specific structure of lagomorphs species living in Poland, with the presence of only domestic rabbits (Oryctolagus cuniculus) and brown hares (*Lepus europaeus*). Wild rabbits living freely, or in captivity, as well as other related species of rabbits (eg. *Sylvilagus floridanus)* do not occur naturally in the environment. Another important aspect is the structure of the rabbit farm with a dominance of mixed-breed rabbits reared in small-scale farms and a relatively few number of industrial farms. The current epidemiological situation regarding RHD and serological status of rabbits has recently been described [12].

The first phylogenetic analysis of Polish RHDV strains collected between 1988 and 2000 (SGM 1988, KGM 1988, LUB 1988, PD 1989, MAL 1994, BLA 1994, GSK 1988, ZD0 2000), was performed in our laboratory in 2003 [10]. These studies were based on the nucleotide sequences encoding the N-terminal fragment of VP60 and a fragment of the p29 gene sequence. Nucleotide and amino-acid sequences from RHDV strains revealed a high genetic identity of isolates and two genetic groups showing temporal similarities were determined. Subsequently, to estimate the genetic variability of Polish RHDV strains and to confirm the presence of the genetic variant RHDVa, the partial nucleotide sequence of the capsid protein gene *VP60,* including two highly variable regions C and E, was determined [11]. Phylogenetic analysis of 15 RHDV strains isolated over a period of 18 years revealed the presence of three genetic groups. The oldest RHDV showed a very high similarity at the amino acid level (98-99%) to the German FRG89 reference strain and to most European viruses isolated in the same period, and also to the Chinese isolate from 1984. The non-haemagglutinating (HA-negative) strains clustered into a second subgroup presenting an intermediate degree of genetic variability (about 3%) in the *VP60* gene fragment. The most genetically variable strains (6-7%) clustered in the RHDVa subtype [11].

The aim of this study was the characterisation of the nucleotide and amino acid sequences of the complete genomes (7.5 kb) of RHDV strains isolated in Poland and an estimation of the genetic variability in different elements of the viral RNA. In addition, the nucleotide sequence of Polish RHDV isolates from 1988-2015 were compared to sequences of other European RHD viruses, including the RHDVa and RHDV2/RHDVb subtypes. Total RNA was extracted from rabbit liver homogenates as described previously [11]. cDNA was synthesized using oligo dT15 and AMV reverse transcription enzyme (Promega). Thirty one pairs of specific oligonucleotide primers targeting different fragments of the RHDV genome were used in alternative combinations to amplify and sequence 33 RHDV RNA fragments. PCR products were analysed on a 1.5% agarose gel, stained with ethidium bromide and visualized with ImageStore documentation system (UVP). Specific PCR products of 434-856 bp from eighteen Polish RHDV strains collected between 1988 and 2015 were obtained (Table 1). The PCR products were gel-extracted and directly sequenced using ABI Prism BigDye^TM^ terminator v3.1Cycle sequencing kit and ABI3773x1 DNA sequencer (Applied Biosystems). The sequence of the full RHDV genome was determined after analysis of partial sequences available in the form of text file (txt) and fluorograms (file.abi) and by the contiguous compilation of these partial nucleotide sequences. This sequence consists of approximately 7428 nucleotides however the first nine nucleotides from the non-translated 5’UTR region of the RHDV genome were not included in the sequence. Identification of this fragment was not always possible because it contains the nucleotide primer sequence. Taking into consideration that this section of the viral RNA genome is relatively stable, it can be assumed that the sequence of the Polish RHDV isolates in this fragment is 100% identical to the sequence of the reference control strain FRG89 and therefore the nucleotide forward primer [22]. Moreover, according to the structure of the RHDV genome [23, 25], the sequence of the first 16 nucleotides from the 5’UTR region is duplicated in genome fragment 5296-5311, i.e. immediately preceding the *VP60* gene [15, 24]. This is why, the position numbering of nucleotides and appropriate ORFs in the complete nucleotide sequence of Polish RHDV isolates has been moved nine positions in relation to the control German strain FRG89. Each RHDV complete sequence contained seven regions of non-structural proteins (NS 1-7), two regions of structural capsid proteins – VP60 and VP10 and a 3’UTR region. For comparison of the nucleotide sequences of the Polish RHDV isolates, reference RHDVs representing the main phylogenetic groups of classical RHDV, RHDVa and RHDV2 as well as the non-pathogenic rabbit lagovirus RCV were examined using BLAST software with blastn [2] and MEGA6 [28] (phylogenetic trees were constructed using the neighbor-joining method). The complete nucleotide sequence of Polish isolates of RHDV has been entered into GenBank. The accession number of the isolates representing the separate genogroups of RHDV, RHDVa, RHDV2 and non-pathogenic virus RCV and RCV-A1 are as follows: FRG 1989 (M67473), AST89 (Z49271), Eisenhuttenstadt 1989 (EF558578), V351 1987 (U54983), BS89 (X87607), SD 1989 (Z29514), Meiningen 1993 (EF558577), Jena 1993 (EF558576), Frankfurt5 1996 (EF558573), Frankfurt12 1996 (EF558572), Triptis 1996 (EF558583), Hartmannsdorf 1996 (EF558586), Rossi 2002 (EF558584), NZ61 (EF558580), N11 (Navarra 2011) (KM878681), CBVal16 (Valpacos 2012) (X96868), Algarve1 2013 (KF442961), RCV-A1 MIC 2007 (EU871528), MRCV 2001 (GQ166866).

**Table 1:** Nucleotide similarity (%) between sequences of 18 Polish RHDV strains as well as representatives from the G1-G6 genogroups, RHDV2, RCV-like (RCV-A1, MRCV) and RHDV FRG89 strains (M67473)/G2

	RHDV strain (year of isolation)	Acc. no.	ORF1				ORF2	CG
			NS1-NS7 (average)	VP60 E I sub-region	VP60 E II sub-region	VP60 (average)	VP10	
			10-5304	6334-6414	6496-6606	5305-7041	7025-7378	10-7437
Polish RHDV strains	KGM (1988)	KP144790	99	99	100	99	99	99
	PD (1989)	KP144789	99	99	100	99	99	99
	MAL (1994)	KU882093	99	99	100	98	99	99
	BLA (1994)	KP144792	94	91	97	96	96	95
	GSK (1998)	KU882092	94	95	95	96	95	94
	ZD0 (2000)	KU882094	94	94	94	95	96	94
	OPO (2004)	KU882094	94	90	97	96	96	94
	GRZ (2004)	KP144791	89	85	90	92	96	90
	KRY (2004)	KY319033	89	87	91	93	97	90
	L145 (2004)	KY679902	89	85	90	92	96	90
	W147 (2005)	KY319035	89	85	90	92	96	90
	STR (2012)	KF677011	88	84	92	92	95	90
	STR2 (2013)	KY679904	88	85	90	92	95	89
	GLE (2013)	KY319032	88	85	92	92	95	90
	RED1 (2013)	KY679903	88	84	89	92	97	90
	SKO (2013)	KY319034	88	85	91	92	95	90
	STR (2014)	KY679905	88	84	91	92	95	91
	BIE (2015)	KY319031	88	84	91	92	95	90
Representatives of RHDV and RCV types	V351 (1987)	U54983	99	99	99	99	99	99
	BS (1989)	X87607	94	94	95	96	96	95
	SD (1989)	Z29514	94	96	95	95	95	94
	AST (1989)	Z49271	94	96	95	96	96	95
	Eisenhuttenstadt (1989)	EF558578	94	96	94	95	96	95
	Meiningen (1993)	EF558577	94	94	95	96	96	94
	Jena (1993)	EF558576	94	94	95	96	96	94
	Frankfurt5 (1996)	EF558573	94	91	95	96	95	94
	Frankfurt12 (1996)	EF558572	94	91	95	96	95	94
	Triptis (1996)	EF558583	89	87	90	93	97	90
	Hartmannsdorf (1996)	EF558586	89	85	91	94	97	90
	Rossi (2002)	EF558584	89	95	96	93	96	90
	NZ61 (2002)	EF558580	97	96	98	98	98	97
	N11 (2011)	KM878681	86	78		82	86	85
	CBVal16 (2012)	KM979445	86	80	80	81	85	85
	Algarve1 (2013)	KF442961	77	78	80	78	85	78
	MRCV (2001)	GQ166866	78	-	-	84	88	80
	RCV-A1 (2007)	EU871528	78	-	-	80	85	78

Similarities are shown for the nonstructural and structural parts of ORF1 and ORF2 of the RHDV genome, as calculated by BLAST (Altschul et al. 1990)

The results of the comparison between nucleotide sequences of eighteen Polish RHDV isolates and the reference strain FRG89 are presented in table 1. This German RHDV strain from 1989 was chosen as the reference due to the similar time and geographical location of isolation. Additionally, eighteen complete sequences representing the main RHDV genetic groups from GenBank were compared. The percentage identity of the complete genome (CG), as well as individual genes encoding the non-structural proteins (NSP 1-7), structural capsid protein VP60, E1 and E2 sub-regions of VP60 encoded by ORF1 and the structural capsid protein VP10 encoded by ORF2 are shown. The overall similarity of the complete sequence of Polish isolates of RHDV reflects the similarity of individual fragments within the genome, except the EI sub-region of the capsid protein where some strains are much more differentiated. The relative identity of the genome sequences of these RHDV isolates confirms a similar evolution pattern amongst RHDV strains, and the probable lack of recombinants. The significant process of recombination involving large fragments of the genome has recently been recorded for RHDV2 viruses in the Iberian Peninsula [21]. On the basis of the results obtained for the complete genome sequences (CG, 10-7437), the RHDV isolates were divided into three groups: the first group consisted of classical RHDV strains (99% sequence identity), and includes the oldest RHDV isolates most similar to RHDV from 90s (KGM, PD and MAL). In the second group were classical RHDVs showing lower genetic similarity (94-95%). This group consisted of four RHDV isolates from 1994-2004, two of which (BLA 1994 and OPO 2004) are non-haemagglutinating strains that show decreased similarity of 90-91% in the EI sub-region. The other two isolates were classified in the phylogenetically youngest genogroup of RHDV: ZD0 2000 characterized by its variable reactivity in the haemagglutination assay and GSK 1998 that was positive in this assay. The third group (90-91% sequence identity) refers to the RHDVa strains from 2004-2015 that demonstrate 84-87% similarity within the nucleotide sequence of the EI sub-region. The nucleotide homology at different regions of the RHDV genome is shown in Table 1. The results of the phylogenetic analysis of the Polish RHDV strains reveals the presence of three classical RHDV genogroups (G2, G4 and G5) as well as the variant RHDVa (G6). Figure 1 presents the dendrogram of the 18 Polish RHDV isolates on the basis of the RHDV complete genome sequence (7.4 kb). The oldest RHDV isolates (KGM 1988, PD 1989 and MAL 1994) clustered into genogroup G2. It can be assumed that the elimination of these strains from the environment occurred at the turn of 1994 and 1995. Genogroup G2 was replaced by the phylogenetically younger strains BLA 1994 and OPO 2004 which clustered into the G4 genogroup, which probably originated from the G3 lineage as represented by the Italian strain BS89. It is noteworthy the significant difference in time of isolation (about 10 years) of the BLA and OPO strains that represent a rare non-haemagglutinating subgroup, considered a phenotypic variant [5]. The last representatives of Polish classical RHDV are isolates GSK 1988 and ZD0 2000 from genogroup G5. A single clade contains the Polish RHDV strains from 2004-2015 (GRZ 2004, KRY 2004, L145 2004, W147 2005, SKO 2013, GLE 2013, RED1 2013, STR 2012, STR2 2013, STR 2014, BIE 2015) identified as RHDVa, which clustered into genogroup G6 as represented by RHDV strain Triptis 1996 and Rossi 2002. In this genogroup three smaller subgroups can be selected (Fig. 1). On the basis of epidemiological data and the results of antigenic and genetic analysis, it can be assumed that the RHDVa variant appeared in Poland at the turn of 2003/2004. In 2004 an epizootic of new RHD outbreaks was observed in Poland, from which haemagglutinating RHDV strains with differing antigen reactions by ELISA assay were isolated [10]. The circulation of RHDV with similar antigenic properties was confirmed repeatedly in 2004-2006. More information about the diagnosis of RHDVa via detection of the *VP60* gene using various molecular techniques was presented previously [6, 10]. Moreover, the antigenic subtype RHDVa was isolated from new RHD outbreaks in Poland in 2012-2016 in the voivodeships podkarpackie, malopolskie and lodzkie (http://www.oie.int/wahis_2/public/wahid.php). The separate clade consisted of RHDV2 strains, which exhibit a greater similarity to the non-haemagglutinating rabbit caliciviruses (Italian RCV, Australian RCV-A1 and American MRCV) [1, 8]. In the last years, the disappearance of classical RHDV strains and the subsequent domination of the RHDVa variant has been observed in Europe, with the exception of the Iberian Peninsula, where until recently the classical RHDV strains from G1 and G5 genogroups were isolated [20]. At present, RHDVa is the dominant virus in Poland; its recognition in RHD outbreaks in 2012-2016 is evidence of continued circulation of this virus in the environment. The spread of RHDV2 in many West European countries and in Scandinavia, Great Britain and Ireland has also been recorded recently [3, 18].

**Fig. 1:**
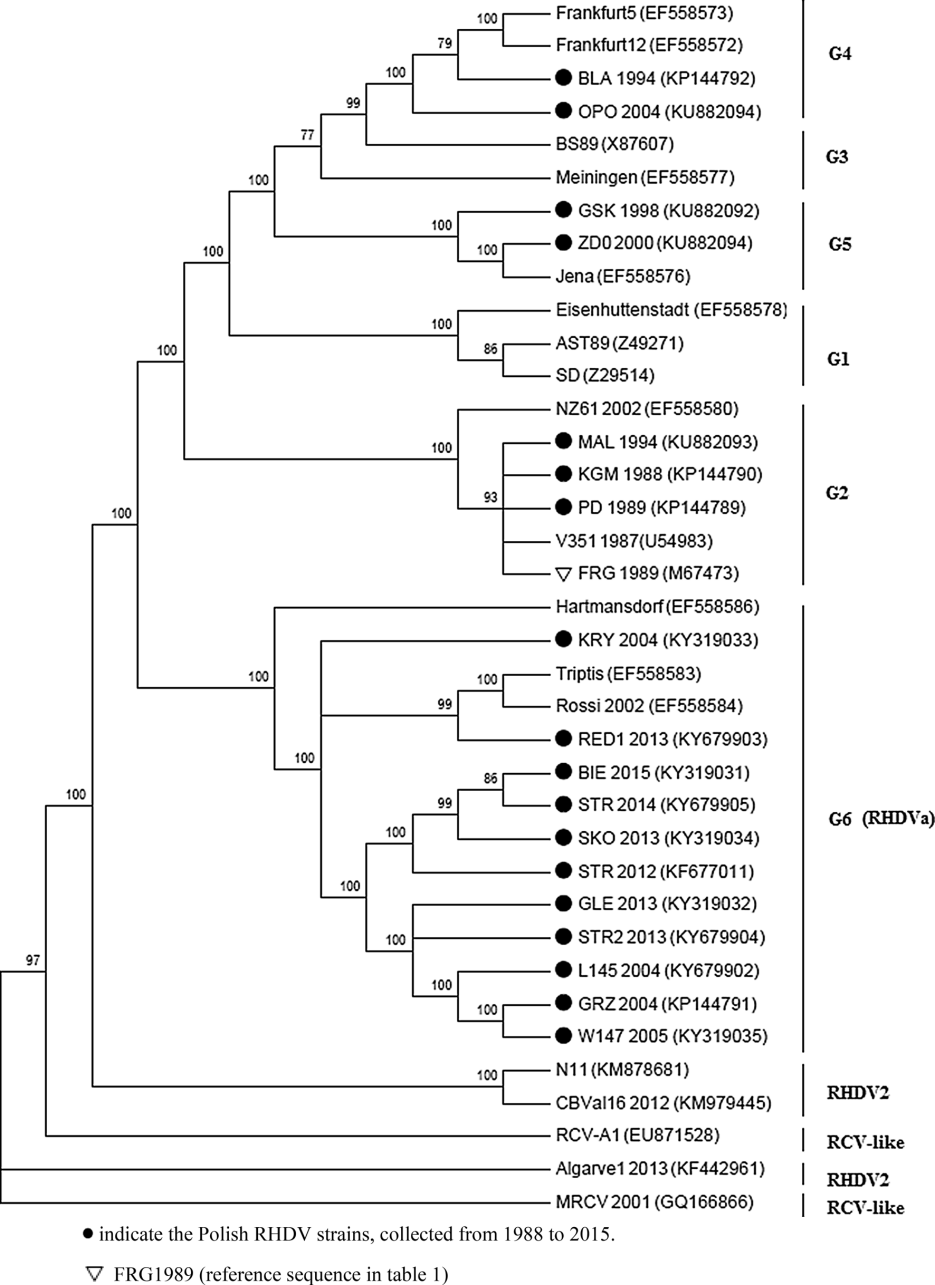
Neighbor-Joining tree of 37 RHDV strains based on CG sequences. The percentage of replicate trees (>70) in which the associated taxa clustered together in the bootstrap test (1000 replicates) are shown above the branches. Evolutionary analyses were conducted in MEGA6 (Tamura et al. [28])

In conclusion, in this study we have reported the complete genome sequence of 18 RHDV strains isolated in Poland between 1998 and 2015. On the basis of comparative analysis of their sequences it can be stated that the genome of these viruses is relatively stable, and although no recombination has been demonstrated the presence of this phenomenon cannot be ruled out. At the end of March 2017, eighteen complete genome sequences were entered in GenBank, of which nine have been published and the next are in the process of verification. The results of sequencing of the oldest Polish isolates from 1988-1994 as well as younger strains from 2012-2015 has confirmed the disappearance of classical RHDV in Poland. Accordingly, all recently isolated RHDV strains belong exclusively to RHDVa; however, no RHDV2 has been diagnosed in Poland.
